# Application of erector spinae plane block and thoracic paravertebral block in thoracoscopic lobectomy

**DOI:** 10.3389/fmed.2025.1740578

**Published:** 2026-01-28

**Authors:** Xiaoqiong Yan, Qin Wang, Li Yang, Tianjing Zhang, Yaru Chen, Lili Zou

**Affiliations:** Department of Anesthesia and Perioperative Medicine, General Hospital of Ningxia Medical University, Yinchuan, China

**Keywords:** erector spinae plane block, postoperative pain, pulmonary space-occupying lesions scheduled, thoracic paravertebral block, thoracoscopic lobectomy

## Abstract

**Objective:**

Compare the effects of erector spinae plane block (ESPB) and thoracic paravertebral block (TPVB) in perioperative pain management for thoracoscopic lobectomy.

**Methods:**

From September 2024 to June 2025, 99 patients with pulmonary space-occupying lesions scheduled for thoracoscopic lobectomy were enrolled and randomly assigned to three groups (33 each): the control group, the ESPB group, and the TPVB group. Baseline data covered gender, age, body mass index, tumor-node-metastasis stage, American Society of Anesthesiologists grade, surgery duration, and resection site. Primary outcomes were resting and coughing visual analogue scale (VAS) scores in the postanesthesia care unit at 2, 4, 8, 16, and 24 h postoperatively. Secondary outcomes included heart rate (HR), mean arterial pressure (MAP), adverse reactions, opioid consumption, time to first ambulation, hospital stay, and drainage tube duration.

**Results:**

Baseline data were comparable. ESPB and TPVB groups had lower VAS scores and area under curve (AUC) than the control group (*p* < 0.05), with TPVB showing the best results (*p* < 0.05). The control group had higher postoperative MAP and HR than the other two groups (*p* < 0.05), while the TPVB group had lower MAP and HR than the ESPB group at 2 and 4 h post-surgery (*p* < 0.05). Adverse reaction rates were similar across groups (*p* > 0.05). The TPVB group also surpassed the ESPB and control groups in opioid consumption, time to first ambulation, hospital stay, and drainage tube duration (*p* < 0.05). The pain level of patients was positively correlated with the time of first getting out of bed, the duration of hospitalization and the duration of drainage tube (*p* < 0.05).

**Conclusion:**

Both TPVB and ESPB can effectively relieve postoperative pain, reduce stress responses, and shorten recovery time after thoracoscopic lobectomy. TPVB offers better early analgesia and hemodynamic stability.

**Clinical trial registration:**

www.chictr.org.cn, Identifier, ChiCTR2100054074, 2021/12/08.

## 1 Introduction

Pulmonary space-occupying lesions refer to a category of diseases characterized by abnormal masses or nodules within the pulmonary parenchyma that occupy the space of normal lung tissue. These lesions typically appear as “shadows” or “masses” on imaging examinations such as chest X-rays and CT scans, with a diameter usually greater than 3 cm, distinguishing them from “pulmonary nodules” that are smaller than 3 cm (1). Surgical resection is the cornerstone treatment for specific types of pulmonary space-occupying lesions. Traditional open thoracotomy has long been the standard surgical approach for lung procedures. However, its significant drawbacks, such as major trauma, severe postoperative pain, long recovery period, and substantial impact on lung function, have spurred the development of minimally invasive techniques. Thoracoscopic surgery has gained prominence due to its notable advantages and has become the preferred surgical method in current clinical practice for early-stage lung cancer and benign space-occupying lesions that require resection. Thoracoscopic surgery involves creating 1 to 3 small incisions, each about 1 to 3 centimeters long, on the chest wall to insert a thoracoscope and fine instruments to complete the resection of the lesion. Its core advantages include minimal surgical trauma, significantly reduced postoperative pain, shorter hospital stays, and better preservation of lung function. However, thoracoscopic lobectomy often induces moderate to severe pain, with some patients even experiencing intolerable agony. If pain is not properly managed, it may trigger a series of short-term pulmonary complications, including impaired cough function, accumulation of respiratory secretions, and reduced functional residual capacity. Additionally, some patients may develop atelectasis and pulmonary infections. About 20 to 40% of patients may experience chronic pain 3 to 6 months after surgery, leading to a decline in quality of life (2). Postoperative pain usually originates from multiple factors, including the stimulation of nociceptors by skin incisions, traction, and stripping of the skin and intercostal muscles during surgical manipulation, as well as injury to intercostal nerves. The intercostal nerves serve as conduits for pain signals, transporting them to the spinal cord before they reach the brain and cause the experience of pain (3). Therefore, effective analgesic measures are crucial for pain management after thoracoscopic lobectomy to promote rapid patient recovery and improve prognosis.

At present, multiple analgesic regimens are used for pain management in thoracic surgery, including thoracic epidural analgesia, systemic opioids, intrathecal opioids, pleural block, thoracic paravertebral nerve block (TPVB), fascial plane block, and intercostal nerve block (4). Thoracic epidural analgesia used to be deemed the benchmark for managing pain during the perioperative period. It can provide effective perioperative analgesia, reduce postoperative pulmonary complications, and shorten the duration of postoperative ileus (5). Nonetheless, thoracic epidural analgesia is associated with certain hazards and constraints, which include the potential for hemodynamic fluctuations, accidental intraspinal injection, the development of epidural hematoma, possible neurological damage, and urinary retention issues (5). With the advancement of technology, ultrasound-guided regional blocks have become an important option for controlling postoperative pain after thoracoscopic lobectomy, due to their advantages of reducing the incidence of complications, decreasing the amount of local anesthetic used, and lowering the risk of nerve injury (6). Among these, TPVB achieves effective analgesia by injecting local anesthetics into the paravertebral space, blocking the ventral and dorsal branches of spinal nerves and sympathetic nerves. This method can produce somatic and sympathetic nerve block of 1 to 3 adjacent segments on the same side as the injection site, similar to unilateral epidural analgesia (6). The erector spinae plane block (ESPB) is a straightforward fascial plane block method. It has the potential to be an alternative to the epidural block and may cause fewer adverse effects. This technique functions by diffusing local anesthetics within the fascial planes, enabling the anesthetics to reach the target nerves and thus providing analgesia (7). However, comparative studies on the role of TPVB and ESPB in pain control during thoracoscopic surgery for patients with pulmonary space-occupying lesions are still relatively rare at present. Given this, the present study aims to explore the differences between TPVB and ESPB in perioperative pain control for thoracoscopic lobectomy, with the goal of providing more effective pain management strategies for clinical practice and optimizing the anesthetic protocol for thoracoscopic surgery in patients with pulmonary space-occupying lesions, thereby improving postoperative recovery quality and quality of life for patients.

## 2 Methods

### 2.1 Study design and participants

**Figure 1:**
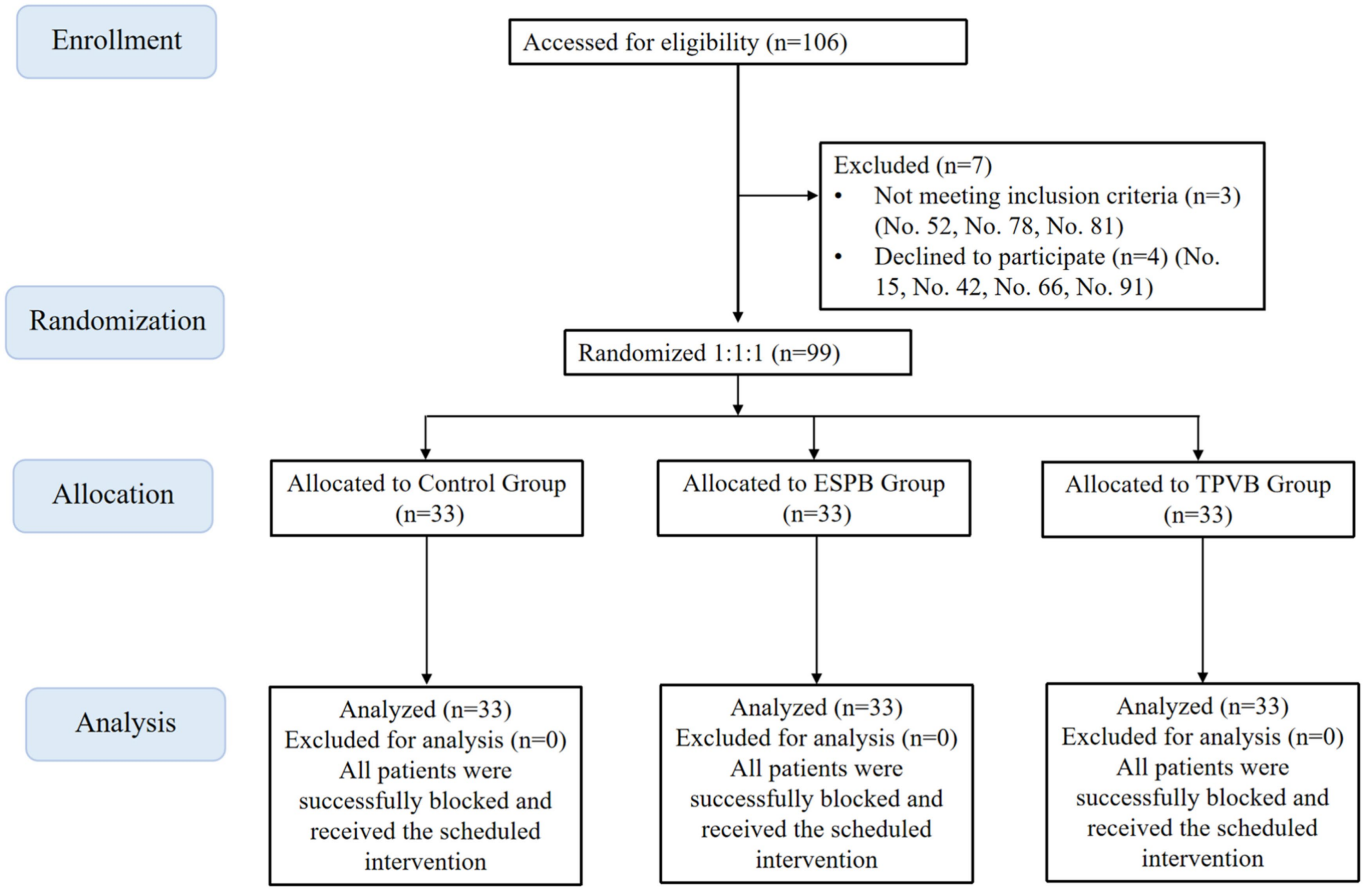
CONSORT flow chart.

Between September 2024 and June 2025, patients with pulmonary space-occupying lesions who needed thoracoscopic lobectomy and fulfilled the diagnostic criteria were recruited from our hospital. Initially, 106 patients were identified. However, four patients were excluded because they declined to take part in the study, and three were excluded due to not meeting the inclusion criteria. Ultimately, 99 eligible patients were selected and evenly divided into three groups at random (Figure 1). The control group, the erector spinae plane block (ESPB) group, and the thoracic paravertebral nerve block (TPVB) group each consisted of 33 participants. The study was approved by the Medical Research Ethics Review Committee of General Hospital of Ningxia Medical University (KYLL-2025-1357). This study has been registered with the Chinese Clinical Trial Center^1^ (No. ChiCTR2100054074, 2021/12/08). All participants provided written informed consent prior to their enrollment in the study.

Randomization was performed using a computer-generated random number sequence. To ensure confidentiality of the randomization, the random allocation sequence was generated by an independent researcher who was not involved in subject recruitment, randomization, or outcome assessment. Subsequently, the random sequence was sealed in sequentially numbered opaque envelopes. After eligible subjects completed the enrollment assessment, researchers sequentially opened the envelopes to determine their group assignment. Blinding: Given the nature of the intervention, blinding of the anesthesiologists performing the procedure was not feasible in this study. However, to minimize bias, the following measures were implemented: All patients remained unaware of their specific group assignment throughout the perioperative period; researchers responsible for postoperative follow-up, data collection, and outcome assessment were completely unaware of group assignments; anesthesiologists aware of group assignments were solely responsible for performing the nerve blocks and did not participate in any subsequent data recording or efficacy assessments. This blinding protocol was effective from the completion of the intervention until the conclusion of the final data analysis.

Diagnostic Criteria: *Guidelines of the American College of Chest Physicians* (ACCP) (8): The radiological features are characterized by homogeneous calcification or smooth margins. Preoperative pulmonary function tests had to meet the following criteria: forced expiratory volume in 1 s (FEV₁) ≥ 60% of the predicted value, a diffusing capacity of the lungs for carbon monoxide (DLCO) ≥ 60% of the predicted value, and an FEV₁/FVC ratio >50%.

Inclusion Criteria: (1) *American Society of Anesthesiologists* (ASA) physical status classification of I to III; (2) Undergoing thoracoscopic lobectomy; (3) 18 ≤ Age<70 years; 4) BMI ranging from 18.5 to 23.9 kg/m^2^. (The establishment of this BMI range aims to control anatomical and pharmacokinetic variations caused by body size differences, thereby reducing confounding bias and ensuring comparability between the two nerve block techniques under standardized conditions.)

Exclusion Criteria: (1) Those with compromised liver or kidney function; (2) Individuals with a history of allergies to local anesthetics; (3) Patients with a diabetic history; (4) Patients with recent use of opioid medications; (5) Patients with abnormal coagulation function or who have not discontinued aspirin for at least seven days; (6) Infection at the puncture site.

Termination Criteria: During the study, if a patient experiences severe adverse reactions that are deemed directly related to the nerve block technique used by a multidisciplinary expert panel, and continuing the study would pose a significant threat to the patient’s life and health, the study will be immediately terminated.

Withdrawal Criteria: If a patient explicitly requests to withdraw from the study for personal subjective reasons during the study process, they will be considered a dropout case. Additionally, if a patient experiences severe unexpected complications during the perioperative period that prevent further pain assessment and monitoring of related indicators according to the predetermined protocol, the patient will also be categorized as a dropout.

### 2.2 Sample size calculation

The sample size was calculated using PASS 15.0 software (NCSS, LLC) based on the data from a pilot study involving 20 patients (not included in the formal trial) who underwent elective thoracoscopic surgery in our hospital in September 2024. The primary outcome was the resting Visual Analog Scale (VAS) score at 24 h post-surgery. The pilot study yielded the following mean ± standard deviation (SD) VAS scores (0–10 cm): Control group: 3.04 ± 0.67 cm; ESPB group: 2.74 ± 0.86 cm; TPVB group: 2.72 ± 0.79 cm.

The calculation was performed for a one-way analysis of variance (ANOVA) model. The effect size (f), calculated from the group means and the pooled standard deviation (0.78 cm), was 0.42, which represents a medium-to-large effect. With a significance level (*α*) of 0.05 (two-sided) and a statistical power (1-*β*) of 80%, the software indicated a minimum requirement of 10 subjects per group.

Accounting for an anticipated 20% dropout rate, the final sample size was adjusted to N = 10/(1–0.20) = 12.5. Therefore, we aimed to enroll at least 13 patients per group, yielding a total target sample size of 39 patients.

### 2.3 Research methods

The control group was subjected to routine preoperative fasting and water deprivation. After entering the operating room, an intravenous access was established, and the patients’ blood pressure, heart rate, and oxygen saturation were monitored. Anesthesia induction was performed using midazolam (specification: 21 mL/25 mg/vial) at 0.05 mg/kg, sufentanil (specification: 2 mL/100 μg/vial) at 0.4 μg/kg, etomidate (specification: 10 mL/20 mg/vial), and rocuronium (specification: 50 mg/vial) at 0.6 mg/kg. Anesthesia was maintained with propofol (specification: 20 mL/200 mg, 5 mg/kg) and remifentanil (specification: 2 mg/vial, 0.3 μg/kg·h). Sufentanil could be appropriately supplemented during the operation to maintain the patients’ Narcotrend index between 40 and 60 (Table 1).

**Table 1:** Comparison of anesthesia management in three groups during and after operation.

Anesthesia management	Control group	TPVB	ESPB
Intraoperative anesthesia management			
Blocking Technique	None	Single injection of TPVB	Single injection of ESPB
Local Anesthetic (intraoperative)	Not applicable	0.375% ropivacaine, 0.15 mL/kg	0.375% ropivacaine, 0.15 mL/kg
Continuous Infusion (intraoperative)	Not applicable	None	None
General anesthesia	The unified regimen was 0.05 mg/kg midazolam, 0.4 μg/kg sufentanil, etomidate and 0.6 mg/kg rocuronium.		
Postoperative Analgesia Management			
Persistent Block	None		
PCA Formula	Sufentanil 2 μg/kg + Normal saline up to 100 mL		
PCA Parameters	Background infusion: 2 mL/h Bolus dose: 0.5 mL Lockout interval: 15 min		
NSAID	Flurbiprofen Axetil 50 mg, IV, every 12 h		
Opioid Rescue Rule	When the resting VAS pain score > 4, nurses may administer Morphine 2 mg IV push. This can be repeated every 10 min until VAS ≤ 4.		

TPVB Group: Ultrasound guidance was provided using the Mindray Anesus ME7 with a C5-1S probe. On the basis of the control group, ultrasound-guided thoracic paravertebral nerve block was added. Before the induction of anesthesia, the puncture point was selected at the side of the affected area, 4–5 cm away from the spinous process under ultrasound guidance, with a frequency of 2–6 MHz. Under ultrasound guidance, after observing the needle advancing to the paravertebral space, aspiration was performed to ensure there was no blood or cerebrospinal fluid. Then, 0.375% ropivacaine hydrochloride at 0.15 mL/kg was injected. After observing the spread of the drug in the paravertebral space, a catheter was placed. The needle was withdrawn, and the catheter was secured. After testing the efficacy of the anesthetic plane, general anesthesia induction was performed. The induction and maintenance of anesthesia were consistent with those in the control group. The postoperative intravenous patient-controlled analgesia measures were also the same as those in the control group (Table 1).

ESPB Group: Patients in the ESPB group received an ultrasound-guided erector spinae plane block before the induction of general anesthesia. The fifth thoracic vertebra (T5) spinous process was identified using the Mindray Anesus ME7 with a C5-1S probe. The probe was positioned 3 centimeters to the side of the T5 spinous process. In the longitudinal parasagittal plane, on the high-echo shadow of the transverse process, the anatomical reference points, namely the trapezius muscle, the major rhomboid muscle, and the erector spinae muscle, were identified. Following disinfection and the application of sterile drapes, a nerve block needle was inserted in-plane from the cranial direction. The insertion continued until the needle tip made contact with the transverse process, which could be visualized beneath the erector spinae muscle. The correct placement of the needle was verified by observing the linear dispersion of 1–2 milliliters of normal saline above the transverse process and below the erector spinae muscle. After negative aspiration, 0.375% ropivacaine hydrochloride at 0.15 mL/kg was injected. After the drug was diffused in the spinal cord, the catheter was inserted, the needle was withdrawn and the catheter was fixed. After the effect of the anesthesia plane was tested, the induction of general anesthesia was performed, the method of induction and maintenance of anesthesia was the same as that of the control group, and the postoperative intravenous analgesia was the same as that of the control group (Table 1).

The anesthetic block level was tested at the anterior axillary line on the affected side in the TPVB and ESPB groups 20 min after nerve block. An ineffective block was classified as a sensory block that covered fewer than three dermatomal segments. Patients with such inadequate blocks were subsequently removed from the study cohort.

In order to guarantee sufficient analgesia during the operation, patients were carefully observed for any indications of insufficient analgesia. Such inadequacy was identified when there was an elevation in heart rate (HR) or mean arterial pressure (MAP) by more than 20% compared to the baseline values. The baseline was determined after the intravenous administration of midazolam and prior to the induction of anesthesia. An analgesia insufficiency event refers to a situation where a patient’s heart rate or mean arterial pressure increases by more than 20% above baseline values after excluding clear causes such as hypovolemia, bladder distension, or drug allergies. This is determined through continuous intraoperative monitoring by the attending anesthesiologist, who is responsible for ruling out other potential causes of vital sign fluctuations and confirming their correlation with surgical stimuli and analgesia insufficiency. The baseline values are defined as the average of three consecutive heart rate and mean arterial pressure measurements taken in a calm state after the patient enters the operating room and before anesthesia induction. Vital signs are non-invasively and automatically recorded every 2.5 min via a multifunctional monitor, with real-time observation by the anesthesiologist during critical surgical procedures (such as skin incision, intercostal expansion, and thoracotomy). These events are classified as follows: Mild events (a 20–30% increase lasting less than 3 min) require continuous monitoring and preparation of rescue analgesics. Severe events (an increase exceeding 30%, or a 20% increase lasting 3 min or longer) necessitate immediate intravenous administration of rescue analgesics like sufentanil (0.1 μg/kg) and evaluation of effectiveness, with formal documentation. All analgesia insufficiency events must be meticulously recorded, including the time of occurrence, peak vital signs, duration, interventions, and post-intervention responses, for inclusion in final data analysis.

After the surgery, extubation was performed following reversal of residual neuromuscular blockade, and the patients were transferred to the postanesthesia care unit (PACU) for 1 h before being transferred to the general ward. According to the analgesic response of the patient, the analgesia was maintained by continuous infusion of 0.125% bupivacaine (8–10 mL/h), and the specific dose and infusion rate were adjusted according to the analgesic response of the patient.

All patients received intravenous flurbiprofen axetil every 8 h. The PACU recorded the hemodynamic parameters and resting VAS scores at 2, 4, 8, 16, and 24 h after surgery in both groups. The VAS scores with cough were recorded at 2, 4, 8, 16, and 24 h. The total morphine consumption within the first 24 h was also recorded. If the patients in any of the three groups experienced pain at any time after surgery, the VAS score was assessed. For VAS scores less than 4, intravenous nonsteroidal anti-inflammatory drugs (ketorolac 30 mg) were administered as adjunctive analgesia; for VAS scores of 4 or more, intravenous morphine was administered, with a maximum dose of 0.1 mg/kg.

### 2.4 Observation indicators

The baseline data of the three groups of patients included gender, age, BMI, TNM stage, ASA grade, duration of surgery, and resection site.

The primary outcome measures were the resting VAS scores of the three groups of patients in the PACU, as well as the resting VAS scores and VAS scores with cough at 2, 4, 8, 16, and 24 h after surgery. This study adopted a minimum clinically important difference (MCID) threshold of 1.5 cm for VAS score, based on the classical validation studies by Jensen et al. (9) and Myles et al. (10).

The secondary outcome measures included HR and MAP levels at 2, 4, 8, 16, and 24 h after surgery, as well as perioperative adverse reactions. The total intraoperative rescue fentanyl consumption, total morphine consumption within the first 24 h after surgery, total postoperative morphine consumption, time to first ambulation, postoperative hospital stay duration, and duration of drainage tube placement were also recorded.

### 2.5 Statistical analysis

Data were analyzed using SPSS 27.0 (IBM Corp., Armonk, NY). Normally distributed continuous data were expressed as $\bar{x}\pm s$, whereas non-normally distributed continuous data were expressed as *M* (*Q_25_*, *Q_75_*). Categorical variables were presented in the form of n(%). For normally distributed quantitative variables, one-way ANOVA was utilized to compare the three groups. Non-parametric statistical methods Kruskal-Wallis test and the Mann–Whitney U test, were employed for analyzing non-normally distributed variables. Group-to-group pairwise comparisons were executed using the independent samples t-test. When analyzing categorical data, the chi-square test was applied. In cases where the expected frequency was fewer than 5, Fisher’s exact test was used instead. To compare the data of different time points in the same group, linear mixed model (LMM) was used. Comparison was performed with the postoperative 8-h time point as the primary endpoint. The correlation between pain severity and postoperative conditions in three groups of patients was analyzed using Spearman’s rank correlation analysis. This study adhered to the intention-to-treat (ITT) analysis principle, with all randomly assigned patients included in their original groups for data analysis. As all nerve block procedures in this study were deemed successful, the per protocol analysis results were fully consistent with the ITT analysis. Statistical significance was defined as a *p* value less than 0.05.

## 3 Results

### 3.1 Comparison of baseline data among the three groups of patients

There was no significant difference between the three groups in terms of gender, age, BMI, ASA classification, duration of surgery, and site of resection (*p* > 0.05, Table 2).

**Table 2:** Comparison of baseline data among the three groups of patients.

Baseline indicator	Control (*n* = 33)	ESPB (*n* = 33)	TPVB (*n* = 33)	F/*χ*^2^	P
Sex				1.122	0.517
Male	19 (57.58%)	20 (60.61%)	23 (69.70%)		
Female	14 (42.42%)	13 (39.39%)	10 (30.30%)		
Age (year)	48.32 ± 9.93	49.77 ± 9.00	47.19 ± 8.19	0.673	0.513
BMI (kg/m^2^)	21.98 ± 1.39	21.77 ± 1.11	21.80 ± 1.26	0.278	0.758
ASA				1.804	0.772
I	22 (66.67%)	18 (54.55%)	17 (51.52%)		
II	7 (21.21%)	10 (30.30%)	10 (30.30%)		
III	4 (12.12%)	5 (15.15%)	6 (18.18%)		
Time of surgery (min)	147.06 ± 19.85	140.44 ± 24.43	148.41 ± 16.87	1.412	0.249
Resection site				2.395	0.966
Right Upper Lung	16 (48.48%)	14 (42.42%)	16 (48.48%)		
Left Upper Lung	10 (30.30%)	9 (27.27%)	9 (27.27%)		
Right Lower Lung	2 (6.06%)	5 (15.15%)	5 (15.15%)		
Left Lower Lung	3 (9.09%)	3 (9.09%)	2 (6.06%)		
Right Middle Lung	2 (6.06%)	2 (6.06%)	1 (3.03%)		

### 3.2 Comparison of VAS scores among the three groups of patients at different time points

VAS scores of the three groups were evaluated in both resting and coughing states (Table 3). Analysis revealed that at various time intervals, the VAS scores for patients in the ESPB group and the TPVB group were notably lower than those of the control group (*p* < 0.05). Furthermore, the TPVB group demonstrated even lower VAS scores compared to the ESPB group (*p* < 0.05). It was also observed that VAS scores during rest were consistently lower than those during coughing (*p* < 0.05). Over time, both resting and coughing VAS scores for all three patient groups exhibited a progressive decline (*p* < 0.05). The control group exhibited significantly higher VAS scores at all time points within 0–24 h compared to the TPVB group (mean difference 1.606,95% CI: 1.410–1.801, *p* < 0.001), with a large effect size (Cohen’s d = 1.82). The second group showed only a marginal advantage (mean difference 0.342,95% CI: 0.174–0.510, *p* < 0.001) and a small effect size (Cohen’s d = 0.39). Time-course analysis revealed a significant decline in VAS values over time, though no significant differences in the magnitude of decline were observed among the three groups (interaction *p* = 0.295) (Tables 4, 5). Compared with the control group, the TPVB group showed MCI in VAS scores at all time points except that no MCI was observed in the 24 h VAS score; the TPVB group and the ESPB group only showed MCI in the indicator of 8 h cough VAS score; while between the ESPB group and the control group, MCI only existed in the 2 h resting VAS score (Table 3). The AUC of VAS scores of resting state and cough in TPVB group and ESPB group were significantly lower than that in control group (*p* < 0.05), and the AUC of VAS scores of resting state and cough in TPVB group was significantly lower than that in ESPB group (*p* < 0.05, Table 6).

**Table 3:** Comparison of VAS scores among three groups of patients at different time points.

Group	VAS at rest						df	P	95% CI
	PACU	2 h	4 h	8 h	16 h	24 h			
Control (*n* = 33)	5.80 ± 1.32	5.82 ± 1.67	5.36 ± 1.22	4.95 ± 1.36	4.37 ± 0.96^a,b,c^	3.63 ± 0.51 ^a,b,c,d,e^	548.151	<0.001	4.799–5.064
ESPB (*n* = 33)	4.51 ± 0.68*	4.21 ± 0.67*^△^	3.91 ± 0.87*	3.61 ± 0.59*^,a,b^	3.32 ± 0.47*^,a,b^	2.43 ± 0.33*^,a,b,c,d,e^	477.246	<0.001	3.548–3.788
TPVB (*n* = 33)	4.08 ± 0.66*^#△^	3.85 ± 0.59*^#△^	3.43 ± 0.62*^#,a△^	3.15 ± 0.54*^#,a,b△^	2.98 ± 0.40*^#,a,b,c△^	2.24 ± 0.27*^#,a,b,c,d,e^	511.561	<0.001	3.201–3.451
F	31.174	30.616	37.974	34.788	39.777	128.263	–	–	–
P	<0.001	<0.001	<0.001	<0.001	<0.001	<0.001	–	–	–

Group	VAS during coughing					df	P	95% CI
	2 h	4 h	8 h	16 h	24 h			
Control (*n* = 33)	7.44 ± 1.50	6.79 ± 1.44	5.84 ± 1.22^b^	5.01 ± 1.07^b,c,d^	4.42 ± 0.76^b,c,d^	471.851	<0.001	91.264–92.585
ESPB (*n* = 33)	6.29 ± 1.17*	5.41 ± 0.79*^,b^	4.62 ± 0.62*^,b,c^	3.95 ± 0.67*^,b,c,d^	3.44 ± 0.54*^,b,c,d,e^	472.670	<0.001	85.659–86.982
TPVB (*n* = 33)	5.77 ± 0.93*^#△^	4.98 ± 0.72*^#,b△^	4.10 ± 0.81*^#,b,c△▲^	3.53 ± 0.51*^#,b,c,d△^	3.01 ± 0.42*^#,b,c,d,e^	471.357	<0.001	81.388–82.708
F	16.215	27.581	31.240	31.222	49.655	–	–	–
P	<0.001	<0.001	<0.001	<0.001	<0.001	–	–	–

*Indicates *P* < 0.05 compared with Control group, # indicates p < 0.05 compared with ESPB group. ^a^indicates *p* < 0.05 compared to PACU, ^b^indicates *p* < 0.05 compared to 2 h postoperatively, ^c^indicates *p* < 0.05 compared to 4 h postoperatively, d indicates *p* < 0.05 compared to 8 h postoperatively, and e indicates *p* < 0.05 compared to 16 h postoperatively. ^△^indicates MCI compared to the control group; ^▲^indicates MCI compared to the ESPB group.

**Table 4:** Test of main effect in LMM.

Item	Effect	F	df	P
VAS at rest	Treatment	139.848	df_1_ = 2, df_2_ ≈ 527.374	<0.001
	Timepoint	143.668	df_1_ = 5, df_2_ ≈ 192.912	<0.001
	Treatment*Timepoint	1.197	df_1_ = 10, df_2_ ≈ 192.912	0.295
VAS during coughing	Treatment	217.470	df_1_ = 2, df_2_ ≈ 472.336	<0.001
	Timepoint	203.622	df_1_ = 5, df_2_ ≈ 163.139	<0.001
	Treatment*Timepoint	7.715	df_1_ = 10, df_2_ ≈ 163.139	<0.001
MAP	Treatment	217.470	df_1_ = 2, df_2_ ≈ 472.336	<0.001
	Timepoint	203.622	df_1_ = 5, df_2_ ≈ 163.139	<0.001
	Treatment*Timepoint	7.715	df_1_ = 10, df_2_ ≈ 163.139	<0.001
HR	Treatment	182.559	df_1_ = 2, df_2_ ≈ 410.096	<0.001
	Timepoint	107.083	df_1_ = 5, df_2_ ≈ 196.672	<0.001
	Treatment*Timepoint	7.842	df_1_ = 10, df_2_ ≈ 196.672	<0.001

**Table 5:** Effect size and 95% confidence interval at the primary time point (8 h) across the three groups.

Item	Comparison	Mean difference	Standard error	95% *CI*	Cohen’s d
VAS at rest	Control vs. ESPB	1.264	0.225	0.823–1.705	1.392
	ESPB vs. TPVB	0.442	0.224	0.003–0.881	0.487
	Control vs. ESPB	1.706	0.226	1.263–2.149	1.879
VAS during coughing	Control vs. ESPB	1.264	0.161	0.947–1.581	1.420
	ESPB vs. TPVB	0.442	0.161	0.125–0.759	0.453
	Control vs. ESPB	1.706	0.161	1.390–2.022	1.919
MAP	Control vs. ESPB	5.448	1.013	3.503–7.473	1.335
	ESPB vs. TPVB	5.646	1.013	3.661–7.631	1.373
	Control vs. ESPB	11.134	1.013	9.149–13.119	2.708
HR	Control vs. ESPB	8.515	1.092	6.376–10.654	1.922
	ESPB vs. TPVB	6.349	1.092	4.210–8.488	1.434
	Control vs. ESPB	14.864	1.092	12.725–17.003	3.355

**Table 6:** Comparison of the AUC of VAS, MAP and HR among the three groups.

Group	VAS at rest	VAS during coughing	MAP	HR
Control (*n* = 33)	112.71 ± 12.73	120.62 ± 13.04	2177.94 ± 50.06	2147.55 ± 57.95
ESPB (*n* = 33)	82.21 ± 5.53^*#^	95.61 ± 7.14^*#^	2036.94 ± 49.86^*#^	1962.64 ± 43.19^*#^
TPVB (*n* = 33)	73.77 ± 5.51^*#^	85.59 ± 6.94^*#^	1923.67 ± 43.47^*#^	1854.67 ± 46.52^*#^
F	186.360	119.764	233.457	293.967
P	<0.001	<0.001	<0.001	<0.001

*Indicates *P* < 0.05 compared with Control group, #indicates *P* < 0.05 compared with ESPB group.

### 3.3 Comparison of MAP and HR among the three groups of patients at different time points

**Figure 2:**
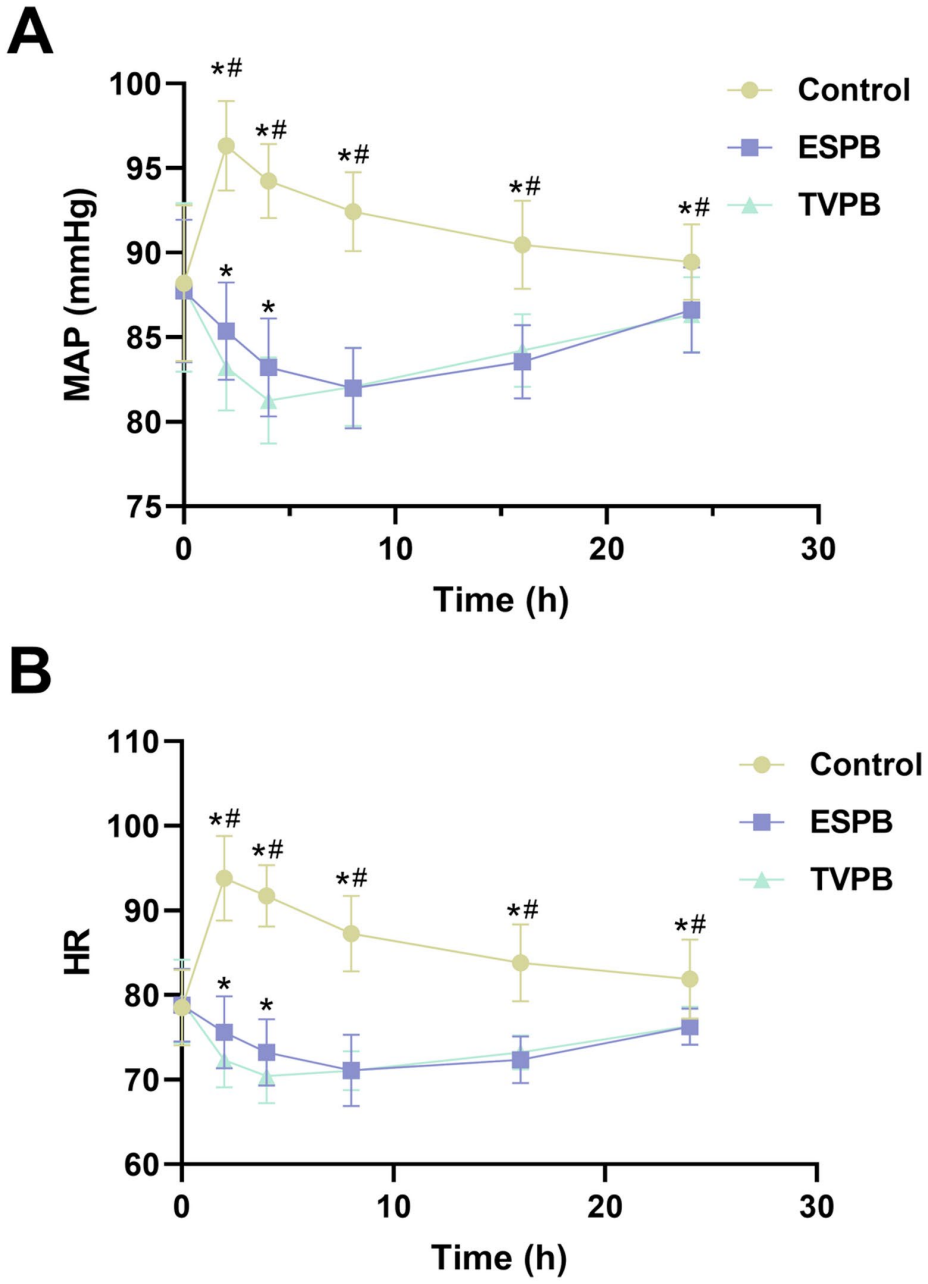
Comparison of MAP **(A)** and HR **(B)** among the three groups of patients at different time points. *Indicates *p* < 0.05 compared with Control group, #indicates *p* < 0.05 compared with ESPB group.

Prior to surgery, no variations in MAP and HR were noted among the groups (*p* > 0.05, Figure 2). Postoperative MAP and HR levels in the control group showed a significant increase and then a gradual decrease, and recovered to close to the preoperative levels at 24 h postoperatively. When compared with the ESPB and TPVB groups, the control group consistently exhibited significantly higher postoperative MAP and HR levels (*p* < 0.05). In contrast, postoperative MAP and HR levels in patients in the ESPB and TPVB groups who underwent nerve block were characterized by a transient decline followed by a slow rebound and a convergence to preoperative baseline levels at 24 h postoperatively. A more detailed comparison revealed that at both the 2-h and 4-h postoperative time points, patients in the TPVB group had significantly lower MAP and HR values than those in the ESPB group (*p* < 0.05). The LMM analysis revealed significant interaction effects between MAP (*F* = 7.715, *p* < 0.001) and HR (*F* = 7.842, *p* < 0.001) across the three groups, indicating substantial differences in temporal effects (Table 4). The primary time point analysis (8 h) showed that MAP and HR in the control group were higher than those in the ESPB and TPVB groups at 8 h (Table 5). Furthermore, the temporal effects of MAP (*F* = 203.622, *p* < 0.001) and HR (*F* = 1.7083, *p* < 0.001) were significant, with the control group exhibiting a trend of initial significant elevation followed by gradual decline, while the TPVB group showed a sustained decrease, and the ESPB group exhibited intermediate trends between the two. The AUC comparison results showed that the AUC of MAP and HR in TPVB group and ESPB group were significantly lower than those in control group (*p* < 0.05), and the AUC of MAP and HR in TPVB group was significantly lower than that in ESPB group (*p* < 0.05, Table 6).

### 3.4 Comparison of perioperative adverse reactions in three groups of patients

Upon comparing the perioperative adverse reactions of the three patient groups, the analysis indicated that, across the postoperative time intervals of 0–1 h, 1–6 h, 6–12 h, and 12–24 h, no significant differences were observed among the groups in terms of hypoxemia, carbon dioxide retention, intraoperative cardiovascular incidents, postoperative respiratory depression, pruritus, dizziness, restlessness, as well as nausea and vomiting (*p* > 0.05, Table 7).

**Table 7:** Comparison of perioperative adverse reactions in three groups of patients.

Perioperative adverse reactions	Control (*n* = 33)	ESPB (*n* = 33)	TPVB (*n* = 33)	*χ* ^2^	P
Hypoxemia	7 (21.21%)	2 (6.06%)	5 (15.15%)	2.996	0.243
Carbon dioxide accumulation	4 (12.12%)	3 (9.10%)	3 (9.09%)	0.325	1.000
Intraoperative cardiovascular events	4 (12.12%)	2 (6.06%)	3 (9.09%)	0.790	0.906
Postoperative respiratory depression	5 (15.15%)	2 (6.06%)	2 (6.06%)	1.945	0.498
Itchy skin	7 (21.21%)	3 (9.09%)	4 (12.12%)	2.017	0.445
Dizziness	7 (21.21%)	2 (6.06%)	3 (9.09%)	3.547	0.223
Agitation	6 (18.18%)	3 (9.09%)	3 (9.09%)	1.576	0.582
Postoperative vomiting					
0–1 h	7 (21.21%)	3 (9.10%)	6 (18.18%)	1.986	0.481
1–6 h	8 (24.24%)	4 (12.12%)	6 (18.18%)	1.616	0.496
6–12 h	5 (15.15%)	4 (12.12%)	2 (6.06%)	1.464	0.614
12–24 h	4 (12.12%)	1 (3.03%)	3 (9.09%)	1.921	0.525

### 3.5 Comparison of intraoperative and postoperative indicators among the three groups

The total intraoperative consumption of rescue fentanyl, total morphine consumption within the first 24 h after surgery, total postoperative morphine consumption, time to first ambulation, postoperative hospital stay duration, and duration of drainage tube placement were compared among the three groups of patients. The analysis revealed that the TPVB group outperformed the ESPB group in all these aspects, and both the TPVB and ESPB groups showed better results than the control group (*p* < 0.05, Table 8).

**Table 8:** Comparison of intraoperative and postoperative indicators among three groups.

Observation indicator	Control (*n* = 33)	ESPB (*n* = 33)	TPVB (*n* = 33)	Z	P
Total intraoperative salvage fentanyl consumption (μg)	189.00 (178.70, 232.10)	115.40 (96.60, 140.00)*	75.30 (61.60, 89.60)*^#^	82.656	<0.001
Total postoperative morphine consumption in the first 24 h of surgery (mg)	23.30 (18.50, 25.40)	11.50 (10.10, 12.60)*	7.40 (6.30, 8.60)*^#^	84.374	<0.001
Total postoperative morphine consumption (mg)	43.40 (36.00, 48.00)	23.00 (17.40, 26.20)*	14.40 (13.10, 17.40)*^#^	78.766	<0.001
First time out of bed (h)	25.80 (20.85, 31.00)	11.70 (10.00, 14.00)*	9.10 (7.85, 11.35)*^#^	72.131	<0.001
Postoperative hospitalization time (d)	7.00 (7.00, 8.00)	6.00 (6.00, 7.00)*	5.00 (5.00, 5.00)*	63.104	<0.001
Drainage tube retention time (d)	6.00 (5.00, 6.00)	4.00 (4.00, 4.50)*	3.00 (3.00, 4.00)*	54.916	<0.001

*Indicates *P* < 0.05 compared with Control group, ^#^indicates *P* < 0.05 compared with ESPB group.

### 3.6 Correlation analysis of pain degree and postoperative condition in three groups

The correlation analysis of pain degree and postoperative condition showed that the pain degree was positively correlated with the first time of getting out of bed, the postoperative hospitalization time, the time of drainage tube insertion and the amount of analgesic drugs (*p* < 0.001, Table 9).

**Table 9:** Correlation analysis of pain degree and postoperative condition in three groups.

Pain indicator	First time out of bed (h)			Postoperative hospitalization time (d)			Drainage tube retention time (d)		
	r	*P*	95% CI	r	*P*	95% CI	r	*P*	95% CI
AUC of VAS at rest	0.762	<0.001	0.661–0.836	0.790	<0.001	0.700–0.856	0.683	<0.001	0.557–0.778
AUC of VAS during coughing	0.701	<0.001	0.580–0.791	0.627	<0.001	0.575–0.788	0.610	<0.001	0.464–0.723

	Total intraoperative salvage fentanyl consumption (μg)			Total postoperative morphine consumption in the first 24 h of surgery (mg)			Total postoperative morphine consumption (mg)		
	r	*P*	95% CI	r	*P*	95% CI	r	*P*	95% CI
AUC of VAS at rest	0.828	<0.001	0.751–0.883	0.787	<0.001	0.696–0.854	0.790	<0.001	0.698–0.855
AUC of VAS during coughing	0.755	<0.001	0.651–0.830	0.790	<0.001	0.699–0.856	0.714	<0.001	0.597–0.801

## 4 Discussion

This study compared the efficacy of ESPB and TPVB in pain management during the perioperative period of thoracoscopic lobectomy in patients with pulmonary lesions, yielding results with clinical implications. The findings demonstrated that both nerve block techniques significantly outperformed traditional anesthesia methods in terms of resting and cough VAS scores at various postoperative time points, with TPVB exhibiting superior analgesic efficacy in most time intervals. Additionally, patients in the ESPB and TPVB groups exhibited more stable postoperative HR and MAP levels. Furthermore, the TPVB group required lower doses of opioid medications during and after surgery, with significantly shorter times to first ambulation, hospital stay, and drainage tube retention duration. However, there were no significant differences in adverse event rates among the three groups. This finding not only further validates the central role of regional nerve block techniques in pain management for thoracic surgery but also provides quantitative evidence for clinicians when selecting individualized analgesia regimens, overcoming the limitations of previously relying solely on operational convenience or theoretical mechanisms to choose anesthetic methods. It opens new perspectives for optimizing perioperative management in patients with pulmonary space-occupying lesions.

The findings of this study demonstrate that across multiple postoperative time frames, both the resting and coughing VAS scores of patients in ESPB and TPVB groups were markedly lower than control group. Additionally, a significant difference was noted, as the VAS scores of patients in the TPVB group were found to be lower than those in the ESPB group. Over the postoperative period, a consistent trend emerged where the resting and coughing VAS scores of patients in all three groups exhibited a gradual reduction. These results align with previous research (11–15), indicating that compared with traditional anesthesia, these two nerve block methods are more effective in controlling postoperative pain. Additional comparisons between the groups revealed that, for the majority of assessment time points, patients in the TPVB group had lower VAS scores than those in the ESPB group (*p* < 0.05), suggesting that TPVB may have certain advantages in alleviating postoperative pain. Notably, the VAS AUC analysis further confirmed this advantage: both the TPVB and ESPB groups showed significantly lower resting and cough VAS AUC values than the control group (*p* < 0.05), with the TPVB group demonstrating a further reduction in VAS AUC compared to ESPB (*p* < 0.05). As a comprehensive indicator of cumulative pain intensity within 24 h postoperatively, the lower VAS AUC in the TPVB group indicates that this technique not only achieves superior immediate analgesia but also provides more sustained control of cumulative pain load. Additionally, this study found that the resting VAS scores were lower than the coughing VAS scores (*p* < 0.05). The reason for this is that the act of coughing causes contraction of the thoracic wall muscles and changes in intrathoracic pressure, which stimulates the surgical incision and surrounding tissues, activating more nociceptors and thereby increasing the pain scores. TPVB, with its broad block effect similar to unilateral epidural analgesia, has a stronger inhibitory effect on the somatic and visceral compound pain triggered by coughing. Although ESPB can also alleviate pain, its block range is limited by the diffusion of the local anesthetic, making it slightly less effective in dealing with the high-intensity pain stimulus of coughing. In terms of mechanism of action, TPVB directly blocks the ventral and dorsal branches of spinal nerves and sympathetic nerves by injecting local anesthetics into the paravertebral space, forming a broad block effect similar to a unilateral epidural block that can act on multiple adjacent segments. This anatomical direct mode of action enables TPVB to more effectively cover the somatic and visceral afferent nerves of the surgical area in thoracic surgery (16). In contrast, ESPB acts on the ventral and dorsal branches of thoracic spinal nerves through the diffusion of local anesthetics in the fascial plane, and its block range and degree are limited by the efficiency of local drug diffusion (17). However, the extent and intensity of its blockage are constrained by the effectiveness of local drug spread. As per clinical investigations, during the crucial 24-h post-thoracoscopy analgesic phase, the average resting VAS scores of the TPVB group were 1.5–2.0 points lower than those of the ESPB group (17). This finding aligns with the analgesic superiority of TPVB observed at most time points in this study, further validating the disparity in efficacy between the two blocking techniques. The outcomes of this research demonstrate that TPVB exhibits comparative advantages in managing early postoperative pain. Meanwhile, ESPB, as a straightforwardly executable fascial plane block method, can also deliver analgesic results surpassing those of conventional anesthesia. It should be noted that this study set the MCID for VAS scores at 1.5 cm, a threshold established based on classical validation standards for pain assessment. Data analysis revealed statistically significant differences in VAS scores between the ESPB and TPVB groups, but mean differences at most time points fell far short of the 1.5 cm MCID threshold. Clinically significant differences were observed only in the 8-h cough VAS scores. This suggests that while statistical analysis supports TPVB’s superior analgesic effect compared to ESPB, this advantage may not translate to perceptible pain relief improvements in most clinical scenarios. In other words, statistical differences do not fully translate into clinical benefits. Therefore, when choosing these two analgesic techniques in clinical practice, we should not only rely on statistical results, but also consider the individual condition of the patient, the specific condition, the complexity and scale of the operation, and the professional level of the operator, so as to avoid overemphasizing the clinical significance of statistical differences.

In terms of intraoperative hemodynamic changes, this study observed significant intergroup differences. The control group exhibited a stress-induced fluctuation pattern of “initial sharp increase followed by gradual decrease” in postoperative MAP and HR, which remained slightly above the preoperative baseline level at 24 h after surgery. In contrast, the ESPB and TPVB groups showed a benign trajectory of “transient suppression - progressive normalization,” both precisely returning to the preoperative baseline value at 24 h after surgery (*p* > 0.05). It should be emphasized that at the 2-h and 4-h time points following surgery, the TPVB group exhibited significantly reduced MAP and HR compared to the ESPB group (*p* < 0.05), demonstrating superior hemodynamic stability. Consistent with the pain assessment results, the AUC analysis of MAP and HR further confirmed TPVB’s superiority in stabilizing hemodynamics: both the TPVB and ESPB groups showed significantly lower MAP and HR AUC values than the control group (*p* < 0.05), with the TPVB group’s AUC being lower than that of the ESPB group (*p* < 0.05). This indicates that TPVB more effectively reduces cumulative hemodynamic fluctuations within 24 h postoperatively, which is closely related to its stronger sympathetic nerve blocking effect. This difference is closely related to the neural regulatory mechanisms of the two block techniques. TPVB achieves direct block of the sympathetic nerve chain of T1-T12 spinal nerves through the diffusion of local anesthetics in the paravertebral space, effectively inhibiting the activation of the sympathetic-adrenal medullary system triggered by surgical trauma. This reduces the release of catecholamines, decreases myocardial oxygen consumption, and lowers peripheral vascular resistance (18, 19). In comparison, although the fascial plane diffusion pathway of ESPB can partially block the dorsal branches of thoracic spinal nerves, its efficacy in blocking presynaptic sympathetic fibers is weaker, resulting in certain limitations in regulating the surgical stress response (20). It is worth noting that none of the patients in the three groups experienced severe hypotension (MAP<60 mmHg) or bradycardia (HR < 50 beats/min) after surgery, indicating that both nerve block techniques are safe for clinical application. However, the advantage of TPVB in hemodynamic control provides a more optimized anesthetic option for lobectomy patients with underlying cardiovascular diseases, and its mechanism may involve the synergistic action of sympathetic nerve block, somatic afferent block, and central stress response inhibition.

During the assessment of perioperative safety, this research revealed that the incidence of postoperative adverse reactions did not show any variations among the three patient groups (*p* > 0.05). This outcome corresponds with the findings of other similar investigations (21, 22), affirming the high level of safety of ESPB and TPVB techniques when applied clinically. From a technical perspective, the precise positioning under ultrasound guidance may be the key factor in ensuring safety. Modern ultrasound visualization technology enables operators to clearly distinguish nerve structures, vascular courses, and fascial layers: TPVB can avoid the pleural reflection and intercostal vessels under ultrasound guidance, accurately inject local anesthetics into the paravertebral space, increase the success rate of unilateral block, and reduce the risk of local anesthetics entering blood vessels or the epidural space (23). ESPB, on the other hand, monitors the spread of local anesthetics in the thoracolumbar fascia space deep to the erector spinae muscle in real time with ultrasound, avoiding nerve injury caused by blind puncture and reducing the incidence of nerve damage (13). No serious local anesthetic toxicity reactions were observed in either group of patients in this study, which may be related to the optimized local anesthetic dosage under ultrasound guidance, reducing the risk of drug accumulation from the source. Although no intergroup differences in adverse reactions were observed in this study, it is still necessary to pay attention to the potential risks of specific complications. TPVB has an extremely low probability of pneumothorax, while ESPB may result in local hematoma, indicating that strict adherence to the layered positioning principles under ultrasound guidance is required in clinical practice (24). Overall, the results of this study support the safe application of ESPB and TPVB in thoracoscopic lobectomy, which is closely related to the progress of modern visualization technology, individualized dose adjustment, and accurate anatomical block.

When comparing crucial perioperative recovery metrics, this research revealed that the TPVB group had a notable edge in promoting rapid recovery. Specifically, the cumulative intraoperative fentanyl rescue dosage and the total 24-h postoperative morphine consumption were significantly lower in the TPVB group than in the ESPB group. Moreover, both the TPVB and ESPB groups consumed significantly fewer opioids compared to the control group (*p* < 0.05). Regarding clinical functional recovery indicators, a progressive improvement was observed. The TPVB group achieved the first ambulation in a shorter time frame compared to the ESPB group. Additionally, both the length of postoperative hospital stay and the duration of drainage tube placement were substantially reduced in the TPVB group. This established a positive feedback loop characterized by “improved analgesia - decreased medication reliance - expedited functional recovery.” Further correlation analysis confirmed that pain severity (AUC of resting and cough VAS) was positively correlated with the time to first ambulation, postoperative hospitalization duration, and drainage tube retention time (*p* < 0.001). This indicates that cumulative pain load is a critical factor affecting postoperative recovery: effective control of cumulative pain can directly promote early ambulation, reduce the risk of long-term bed-related complications, and consequently shorten hospitalization duration and drainage tube retention time. In contrast, the relatively higher cumulative pain in the ESPB group and control group may delay the initiation of rehabilitation activities and prolong the recovery process. This difference is directly related to the profound analgesic effect of TPVB. The strong block effectively inhibits central sensitization triggered by surgical trauma, while patients in the ESPB group often need to delay similar rehabilitation activities until 12 h after surgery due to the relatively limited block range (25). Moreover, the reduction in opioid consumption not only decreases drug-related complications such as nausea, vomiting, and respiratory depression but, more importantly, avoids *μ*-opioid receptor activation-induced ileus, creating conditions for early enteral nutrition and ambulation (26, 27). The shorter duration of drainage tube placement may be related to two factors: first, TPVB alleviates pleural pain caused by drainage tube irritation by blocking intercostal nerve afferents, enhancing patients’ tolerance to the tube; second, stable hemodynamics reduce postoperative bleeding in the pleural cavity, providing a safe basis for early tube removal (28, 29). Although the ESPB group was inferior to the TPVB group in recovery indicators, it was still significantly better than the control group, indicating that fascial plane block techniques have an undeniable basic analgesic value in thoracic surgery, especially applicable in clinical scenarios with anatomical variations in the paravertebral space or insufficient operational experience.

In conclusion, both ESPB and TPVB serve as effective postoperative analgesia methods for thoracoscopic lobectomy, effectively alleviating pain, reducing stress responses, and shortening recovery time. Compared to ESPB, TPVB demonstrates more significant advantages in mitigating postoperative cumulative pain (lower VAS AUC), stabilizing hemodynamic fluctuations (lower MAP and HR AUC), and promoting recovery. The positive correlation between pain load and recovery metrics further supports this conclusion. Nevertheless, this study still has some limitations: First, the sample size is relatively small, and all participants came from a single center, which may introduce selection bias; at the same time, the BMI range of participants was limited to 18.5–23.9 kg/m^2^, which, while enhancing the internal validity of the study, also restricts the generalizability of the results to overweight or obese populations. Therefore, the conclusions of this study are primarily applicable to normal-weight individuals. Second, this study only observed pain conditions and related indicators within 24 h after surgery, and the evaluation of long-term analgesic effects and patient outcomes is insufficient. Future studies need to expand the sample size, conduct multicenter studies, and include more representative patient populations for long-term follow-up to verify the generalizability, long-term effectiveness, and broader applicability of the study results.

## Data Availability

The original contributions presented in the study are included in the article/supplementary material, further inquiries can be directed to the corresponding author.

## Footnotes

1
www.chictr.org.cn
